# Shedding Light on the Past: Temporal Classification of Zoological Specimens from Museum Collections with Portable NIR Sensors and Multivariate Error Modeling

**DOI:** 10.1021/acs.analchem.5c06767

**Published:** 2026-03-20

**Authors:** Jordi Riu, Barbara Giussani, Manuel Monti, Lorenzo Baruffaldi, Marc Campeny, Javier Quesada

**Affiliations:** † 16777 Universitat Rovira i Virgili, Department of Analytical Chemistry and Organic Chemistry, Carrer Marcel·lí Domingo 1, Tarragona 43007, Spain; ‡ Dipartimento di Scienza e Alta Tecnologia, 226462 Università degli Studi dell’Insubria, Via Valleggio 9, Como 22100, Italy; § 5779 Museu de Ciències Naturals de Barcelona, Castell dels Tres Dragons, Passeig Picasso s/n, Barcelona 08003, Spain

## Abstract

Museum scientific collections preserve invaluable biological archives that provide insights into historical biodiversity and environmental change. Determining the age of specimens often relies on destructive, labor-intensive, and costly methods, limiting their use on rare or valuable materials. In this study, we present a fully nondestructive and rapid approach for classifying the temporal origin of zoological skeletal specimens using portable near-infrared spectroscopy combined with an advanced chemometric framework, exemplified by red squirrel (*Sciurus vulgaris*) bones. Two compact NIR instruments, covering distinct wavelength ranges, were employed to analyze bone samples collected from two temporal groups: “historical” (1916–1923) and “modern” (2005–2021). To extract chemically meaningful information while accounting for instrumental and physical variability, we implemented a maximum likelihood principal component analysis–logistic regression (MLPCA-LR) strategy that explicitly incorporates the measurement error covariance structure. The resulting models achieved perfect or near-perfect discrimination, validated through cross-validation, independent test sets, and bootstrap analysis. Compared to the widely used partial least-squares discriminant analysis (PLS-DA), the MLPCA-LR framework demonstrated superior robustness and interpretability. This study suggests that NIR spectroscopy with portable sensors, combined with MLPCA-LR, offers a nondestructive and accessible approach for temporal classification of skeletal specimens, enabling practical in situ screening in museums without invasive sampling or expert operators.

## Graphical abstract

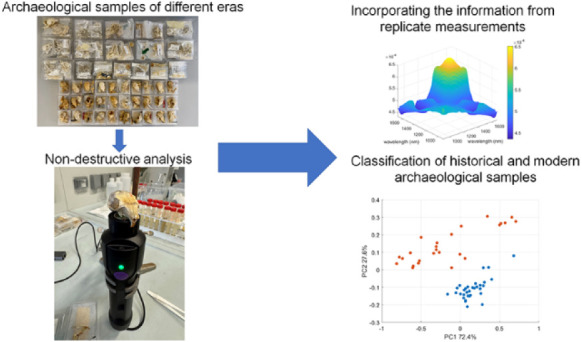

## Introduction

Scientific collections preserved in natural sciences museums provide a direct window into historical biodiversity, environmental changes, and genetic evolutionary trends.^1,2^ Of particular interest are historical specimens housed in zoological collections, which are invaluable for reconstructing past biological processes and understanding long-term ecological and evolutionary patterns.^3^ However, to maximize their scientific utility, these specimens must be accompanied by comprehensive biological and historical metadata, including species identification, sex, age, and—critically—the date and location of collection. Among these, determining the exact date of collection poses a significant challenge, especially when documentation is incomplete or missing.^4^ It is currently estimated that approximately 120 million specimens worldwide cannot be used in research due to the absence of temporal information.^4^ The ability to rapidly and accurately ascertain the age of specimens is crucial for a wide range of studies, yet traditional dating methods such as amino acid racemization, radiocarbon dating or Pb-210 dating, among others, are often time-consuming, costly, and typically require destructive sampling, which is frequently unacceptable for rare or valuable specimens.^5^ This context highlights the urgent need for nondestructive analytical techniques that can provide reliable chronological information directly in a museum setting. To this end, spectroscopic techniques represent a promising approach, as they have the potential to fulfill most of these requirements.

Vibrational spectroscopy^6^ has been recently applied to the study of skeletal remains, both human and nonhuman. For instance, near-infrared (NIR) spectroscopy, either alone^7,8^ or combined with hyperspectral imaging,^9,10^ has been used for the analysis of bones. NIR was used to classify bones from different animal species, such as mammalian, avian, and reptile.^11^ However, some applications have required invasive procedures; for example, Schmidt et al.^12^ used eight hand-held NIR instruments to classify different postmortem intervals (PMI) of human skeletal remains, but the analysis involved cutting a transversal section from each bone. Similarly, Fourier transform infrared (FTIR) spectroscopy has been employed in various studies of skeletal remains, but these analyses have typically been destructive, either because the bones were cut or ground to powder. Examples include its use in general diagenesis studies,^13^ for determining bone mineralization indexes,^14,15^ and to differentiate between buried and unburied human bones.^16^ Raman spectroscopy, sometimes in conjunction with FTIR, has also been utilized to evaluate the PMI of skeletonized remains^17,18^ and to differentiate between present-day and ancient bones from burned skeletal remains, which required acid treatments.^19^ The estimation of the PMI of human skeletal remains has also been carried out with other sophisticated techniques such as microcomputed tomography, mid-infrared microscopic imaging, and energy dispersive X-ray mapping,^20^ which also involved invasive analyses that included cutting the bones. More recently, Raman spectroscopy has been used for PMI prediction in human skeletal remains, again relying on invasive sample preparation.^21^

This study emphasizes the development of nondestructive, sample-preserving methodologies to distinguish the temporal origin of specimens, a critical consideration when working with valuable scientific collections. Specifically, we focus on classifying red squirrel (*Sciurus vulgaris*) bones into two predefined temporal groups: a “historical” group (collected between 1916 and 1923) and a “modern” group (collected between 2005 and 2021). These specimens were selected because the Natural Sciences Museum of Barcelona (NSMB) holds a relatively large number of bones with precisely known collection dates, providing an ideal data set for testing and validating methodological approaches.

To tackle this complex problem, we selected NIR spectroscopy, a nondestructive technique. The analysis was performed using two different portable and cost-effective NIR instruments covering distinct wavelength ranges, with almost no sample preparation. The use of portable instrumentation is a key advantage, as it makes this technology more accessible and allows for in situ studies within institutions such as museums, obviating the need to transport fragile and valuable specimens to an external laboratory. The actively developing field of miniaturized NIR spectroscopy is making inroads into the portable instrumentation market,^22^ a trend characterized by analytical strategies and measurements that vary significantly between instruments and sample types, each with its own set of pros and cons.^23^

The application of chemometrics has proven to be a valuable tool for addressing challenges related to cultural heritage and archeology,^24^ and to analyze the complex data generated by these instruments we propose a novel and robust chemometric framework. Instead of relying on conventional data preprocessing techniques to handle instrumental data, this study leverages the multivariate error structure of the data. By first estimating the error covariance matrix (ECM) from replicate measurements, we employ maximum likelihood principal component analysis (MLPCA), an advanced dimensionality reduction technique that directly incorporates this error information to isolate the relevant chemical information from measurement noise. The resulting information-rich scores are then used to build a classification model using logistic regression (LR). To ensure the reliability of this MLPCA-LR approach, the developed models were rigorously validated^25^ using three distinct methods: cross-validation, prediction on an external test set, and bootstrap analysis. Finally, the performance of this framework was benchmarked against that of the widely used partial least-squares discriminant analysis (PLS-DA) method.

It is important to acknowledge that the scope and generalization capability of this classification strategy are inherently tied to the nature of the reference collection. Since chemometric models are strictly data-dependent, their applicability relies on the training set. While the methodology itself is broadly applicable, the resulting models will be most general when built upon sufficiently large, well-documented, and representative data sets.

## Materials and Methods

### Zoological Samples

A total of 59 red squirrel (*Sciurus vulgaris*) specimens from the collection of the NSMB collected through Catalonia were analyzed. The samples were prepared following procedures that have remained essentially unchanged for nearly a century^26,27^ and stored under stable, dust-free conditions and later in neutral methacrylate boxes, with studies indicating no harmful acidity, suggesting that storage had minimal or negligible impact on bone properties.^28^ For each individual, the animal’s skull (either complete or in fragments large enough to be measured) and at least one complete mandible were available. All specimens, dated between 1916 and 2021, were divided into two groups: 33 “historical” (1916–1923) and 26 “modern” (2005–2021) specimens. Figures S1–S5 in the Supporting Information shows some pictures of the specimens.

### NIR Sensors

#### NeoSpectra Scanner

The NeoSpectra Scanner (Scanner—Si-Ware, Cairo, Egypt) is a portable instrument (1350–2550 nm). It employs a tungsten-halogen lamp, a MEMS Michelson interferometer, and an InGaAs photodetector. Operated via Bluetooth with a proprietary mobile app, the battery-powered instrument acquired each 5 s spectrum without interpolation. Calibration was carried out prior to each measurement using a >99% reflectance Spectralon standard and a scan time of 5 s was used. Spectral data (“.Spectrum” format) were converted to Excel files and processed in MATLAB R2024b (MathWorks Inc., Natick, MA, USA) with PLS_Toolbox Version 9.5.0 (Eigenvector Inc., Manson, WA, USA). The instrument is capable of operating on battery power.

#### VIAVI MicroNIR On-Site W

The VIAVI MicroNIR On-Site W (MicroNIR—VIAVI Solutions, CA, USA) is a hand-held instrument (908–1676 nm) featuring dual tungsten-halogen lamps and a linear variable filter. Operated wirelessly (Bluetooth) via PC/tablet with proprietary software, the battery-powered device scans in <2 s. The background on the VIAVI MicroNIR On-Site W was corrected using a dark reference to account for detector noise and a white reference to normalize sample reflectance as suggested by the manufacturer. Integration time of 0.1 ms and scan count of 100 were used.

### Sample Analysis

Bone surfaces were gently wiped with a water-dampened cotton swab to remove dust and then air-dried. Preliminary assessments identified the frontal bone (skull) and the mandible as the most suitable regions, the former for spectral stability (lower standard deviation) and the latter for its consistent presence in collections. To account for surface heterogeneity, five replicates were acquired from each sample with repositioning, as previous studies confirmed that sample variance significantly exceeds instrumental.^29,30^ To ensure real-world applicability, measurements were conducted under natural environmental variability (ambient temperature and humidity fluctuations) at different times of day across four sessions per instrument, with three different operators and recalibrations, ensuring the resulting MLPCA-LR models are robust to typical procedural and environmental variations during routine collection screening.

Spectra were collected in contact mode, placing the sample on the vertical instrument without pressure to avoid damage as shown in Figure 1. All parts of a specimen were analyzed within the same session and balancing the total sample load across sessions.

**Figure 1:**
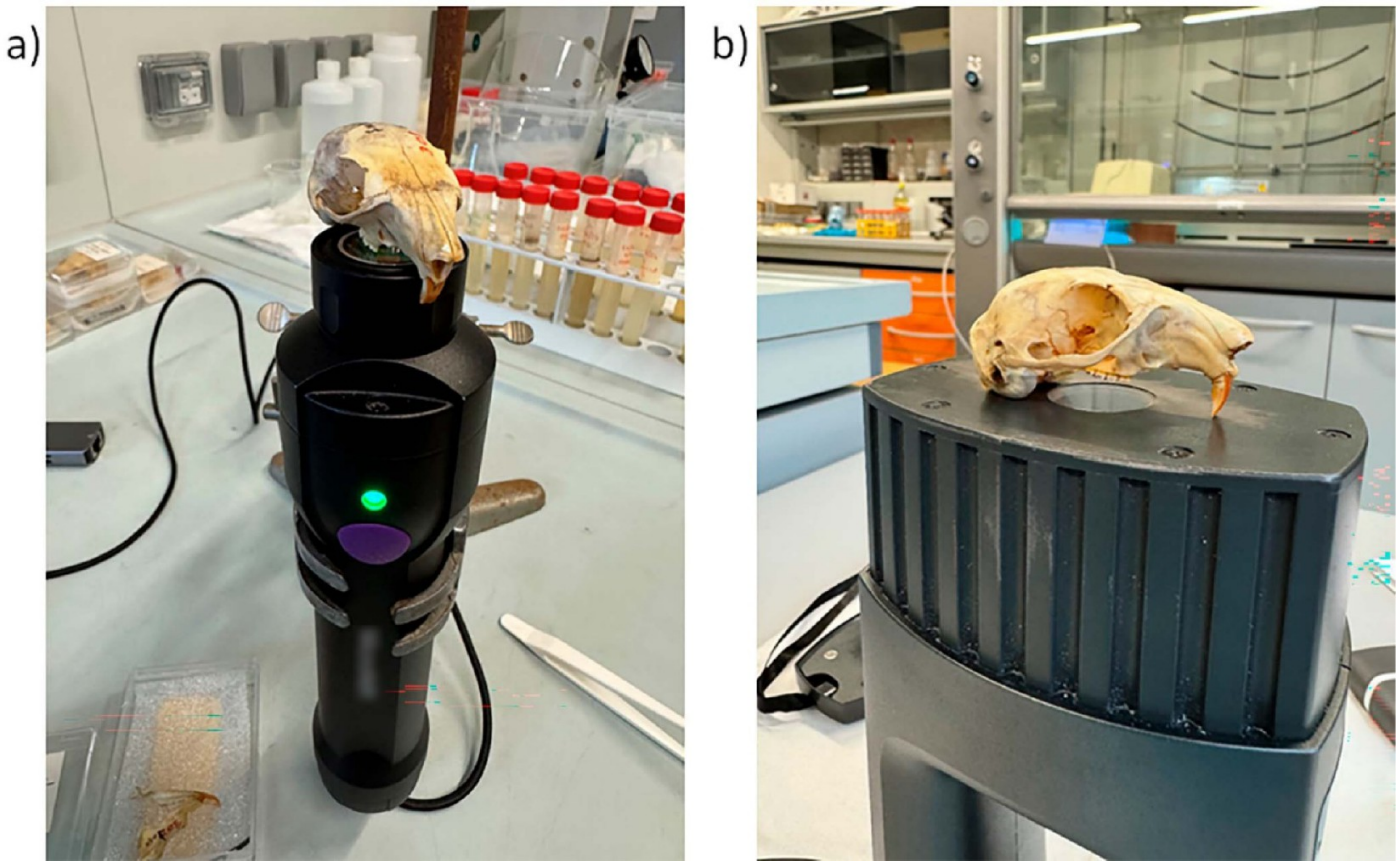
Measurement using a) VIAVI MicroNIR On-Site W and b) NeoSpectra Scanner. In the final protocol, samples were rotated so that the flat part rested against the window.

Lettering on the bones did not affect the spectra. As samples did not completely cover the measurement window, surface area effects are expected in the multivariate analysis. The analytical protocol was designed to be straightforward and transferable for reliable implementation by nonexperts in museums.

### Chemometric Data Treatment

#### Error Covariance Matrix (ECM)

The ECM^31^ represents a widely used method for evaluating multivariate measurement errors, as it elucidates the structure of error correlations between different channels or wavelengths.^32^ The ECM is a symmetric matrix where the diagonal contains the error variance for each channel, and the off-diagonal positions contain the error covariance between channel pairs. A visual inspection of the ECM yields significant insights, disclosing the nature of the errors (such as constant or proportional patterns) and highlighting whether they are uniform across the spectrum (homoscedastic) or variable (heteroscedastic).^33^ The ECM can be estimated through various methodologies, primarily the theoretical, empirical, and experimental.^31^ Given limited a priori information, as in this miniaturized NIR analysis of real samples, the experimental method is preferred as it relies on sufficient replicate measurements (*r*). This procedure involves using the mean of the replicates (*X̅*) as an estimate of the “true” spectrum, from which residuals are calculated by subtracting this average from each scan (*X*). The ECM (Σ_*cov*_) is then computed as the covariance of these resulting residuals:

1 $$\Sigma_{cov}=\frac{\overset{r}{\underset{k=1}{\sum }}{\left(X_{k}-\mathrm{X̅}\right)}^{T}\cdot \left(X_{k}-\mathrm{X̅}\right)}{r-1}$$

Two main issues emerge from this experimental approach. The presence of a few channels with very high variance can mask subtle error relationships in the ECM’s visual representation. This is addressed by calculating the error correlation matrix (Σ_*corr*_), which normalizes the data to present a scale-free view of the error structure with values bounded between −1 and 1:

2 $$\Sigma_{corr}=\Sigma_{cov}/\sqrt{diag\left(\Sigma_{cov}\right)\cdot diag{\left(\Sigma_{cov}\right)}^{T}}$$

Estimations derived from a small number of replicates can suffer from considerable uncertainty. To obtain a more robust estimate, it is common practice to pool the ECMs by averaging them across various sample subsets,^34^ a technique that is especially well-suited for near-infrared data where spectra from similar samples tend to be highly consistent. In this case errors were pooled according to the type of sample (skull or mandible).

#### Maximum Likelihood Principal Component Analysis (MLPCA)

MLPCA^35–37^ is a statistically rigorous alternative to conventional principal component analysis (PCA) for data exploration. MLPCA is an advanced subspace modeling technique that integrates multivariate error information directly into its decomposition procedure to yield more robust and accurate solutions. MLPCA operates by minimizing an objective function that uses multivariate error information to weigh the residuals:

3 $$S^{2}=\overset{n}{\underset{i=1}{\sum }}\left({\mathrm{X̅}}_{i}-{\mathrm{X̂}}_{i}\right)\Sigma_{cov,i}^{-1}{\left({\mathrm{X̅}}_{i}-{\mathrm{X̂}}_{i}\right)}^{T}$$

where *n* is the number of samples (typically each sample *X̅*_*i*_ is the average of *r* replicates) and *X̂*_*i*_ is the data estimated by a *p*-dimensional model. Unlike standard PCA, which minimizes the sum of squared orthogonal distances from the data to the model plane, MLPCA minimizes a function where residuals are weighted according to their corresponding ECM. This ensures that observations with higher uncertainty have less influence on the final model.

MLPCA is not a single algorithm but rather, as defined by P.D. Wentzell,^37^ a framework of different implementations (cases A–F), depending on the error structure of the data matrix. The specific cases address various forms of heteroscedastic and correlated errors,^37^ which are case A: homoscedastic uncorrelated errors (MLPCA is equivalent to PCA); case B: heteroscedastic (common row or column structure) uncorrelated errors; case C: heteroscedastic (random structure) uncorrelated errors; case D: correlated errors, row or column only with common structure; case E: correlated errors, row or column only with unrelated structures; case F: correlated errors, fully correlated measurement errors. The computational approach for solving the model depends on the complexity of the error structure; simpler cases (A, B, and D) can be solved directly, while the more complex cases (C, E, and F) require iterative optimization. In this study, a globally pooled ECM—calculated from the joint information on all specimens (both historical and modern)—was applied to all samples. MLPCA algorithm D on average spectra were used in this research.

#### Logistic Regression (LR)

MLPCA scores were used to construct an LR classification model. Scores were first autoscaled, preventing high-variance components (e.g., PC1) from dominating the coefficients. This allows the regression to determine importance based solely on discriminative power.

LR^38,39^ is a statistical method modeling the relationship between an **X** matrix (in this case the autoscaled scores) and a dichotomous response variable (0/1, corresponding to each one of the selected classes). The model predicts the logarithm of the odds (log-odds, also known as the logit), rather than the class label. This quantity (the log-odds) is derived by first calculating the odds—defined as the ratio of the probability of class membership (*P*_*i*_ for the *i*th sample) to the probability of nonmembership (1 – *P*_*i*_)—and subsequently taking its natural logarithm. This transformation maps the probability, which is bounded between 0 and 1, onto an unbounded scale (negative to positive infinity). This unbounded logit is then modeled as a linear combination of the predictor variables, defined by β coefficients, which equal the number of MLPCA components plus an intercept (β_0_):

4 $$\log\left(\frac{P_{i}}{1-P_{i}}\right)=\beta_{0}+\beta_{1}\times t_{i,1}+\beta_{2}\times t_{i,2}+...+\beta_{p}\times t_{i,p}$$

where *t*_*i*,*k*_ is the autoscaled score of the *i*th sample on the *k*th PC of a *p*-dimensional MLPCA model. The β coefficients are estimated using the principle of maximum likelihood estimation,^38,40^ which seeks the values that make the observed data most probable. Once these coefficients are determined, the linear combination (the log-odds) is transformed back into a probability via the logistic (or sigmoid) function. Finally, class assignment is determined by applying a decision threshold (typically 0.5) to this probability.

For classification, the “historical” class (primary interest) was assigned “1” (the positive class) and “modern” “0”. This standard logistic regression coding allows the model to estimate the probability of the positive class (P­(*Y* = 1|*X*)), ensuring parameters and performance metrics are directly interpretable relative to this primary outcome.

The optimal number of PCs to include in the LR model was determined through 10-fold stratified cross-validation on the training set. The selection criterion was based on the principle of parsimony, choosing the model with the minimum number of PCs that yielded the highest classification performance to ensure generalization and avoid overfitting.

#### Partial Least-Squares Discriminant Analysis (PLS-DA)

Data were also classified using PLS-DA, a widely used comparison method well-suited for two-class problems. Unlike MLPCA-LR, PLS-DA does not incorporate information from the multivariate error covariance matrix during dimensionality reduction. Consequently, its performance is critically dependent on suitable preprocessing.

The average of the spectra recorded for each sample was used to build the **X** matrix containing information about the instrumental responses, and a ***y*** vector was used containing dummy response values related to the two selected classes (“1” for the class “historical” and “0” for the class “modern”).

#### Model’s Validation and Statistical Data Analysis

##### Data Set Partitioning

The full data set was randomly partitioned into a training set of 40 samples (22 historical, 18 modern) and an external test set of 19 (11 historical, 8 modern). This partitioning was performed before any analysis, ensuring the test set remained unseen. Consequently, all model building and internal validation steps (including bootstrap) were performed solely on the training set.

##### Cross Validation (CV)

Models were internally validated using 10-fold random stratified cross-validation on the training set to determine the number of factors. The set was partitioned into 10 segments, preserving the original class proportions in each.

##### External Validation with a Test Set

Following the internal validation, the final models were validated on the external test set to assess its generalization performance on unseen data.

##### Bootstrap

Bootstrap^41^ is a computational resampling procedure for estimating the precision associated with a model’s parameters. The technique operates by repeatedly drawing new sample sets with replacement from the original data and re-estimating the model of interest for each set. This process yields a distribution of the model parameters from which their precision can be inferred, conceptually similar to how the precision of a result is estimated from replicate analyses of a sample. To evaluate the stability of the model-building process based on the training data, bootstrap resampling was confined exclusively to the training set described above, to preserve the integrity of the external test set for a final, independent model evaluation.

In each bootstrap iteration, an MLPCA-LR classification model is built using the selected bootstrap samples. Due to sampling with replacement, certain data points are not selected in each iteration; these are referred to as “out-of-bag” (OOB) samples.^42^ These OOB samples are then classified using the MLPCA-LR model built in that iteration, and a set of performance metrics can be calculated. By repeating this process a large number of times (1000 iterations in this work), the average value and standard deviation for each classification metric can be determined from the 1000 individual values.^43^

Bootstrap validation was used beyond performance assessment to quantify the precision of the figures of merit. We estimated stability by calculating the metrics’ standard deviation over 1000 iterations. A robust model yields strong performance with low variability, indicating the results are not highly dependent on the specific training samples.

##### Quality Parameters Used for Assessing the Goodness of the Classification

Accuracy, sensitivity, specificity, precision, negative predictive value (NPV), false positive rate (FPR), false negative rate (FNR) and the area under the curve (AUC) were the parameters used to assess the goodness of the classification.^44^

#### Statistical Data Analysis

MATLAB 2024b (MathWorks Inc., Natick, MA, USA) with the Statistics and Machine Learning Toolbox, and PLS_Toolbox 9.5.0 (Eigenvector Inc., Manson, WA, USA) for MATLAB were used for data analysis. MLPCA and LR were performed with MATLAB and the Statistics and Machine Learning Toolbox and PLS-DA was performed with the PLS_Toolbox. The MLPCA models were computed using algorithms for MATLAB developed by the research group of Professor P.D. Wentzell (Dalhousie University, Canada), freely available for download: http://groupwentzell.chemistry.dal.ca/software.html. Different spectral preprocessing methods were tested for PLS-DA: multiplicative scatter correction (MSC), standard normal variate (SNV) and first and second Savitzky–Golay derivatives with a different number of smoothing points (from 7 to 21 points). After spectral preprocessing, data were mean-centered.

## Results and Discussion

### Spectroscopic Data Acquisition and Spectra Interpretation

Figure 2 shows the average spectra of all the samples. The NIR spectra shown in Figure 2 reflect the main chemical components of bone: a protein matrix (collagen),^45^ mineral content (hydroxyapatite, which has O–H groups),^46^ water, and lipids (fats).^47,48^ The observed bands are broad and overlapping, which is typical for solid biological samples. The following absorption bands can be identified across the spectra from both instruments:^8,49–52^

- around 970 nm (MicroNIR): small, broad feature corresponds to the second overtone of the O–H stretching vibration from water.
- around 1200 nm (MicroNIR): second overtone of C–H stretching vibrations. In bone, this signal may originate from both the organic protein matrix (collagen, from its amino acid side chains) and lipids (from fatty acids).
- 1450–1500 nm (MicroNIR and Scanner): major absorption system probably resulting from the overlap of two strong bands, the first overtone of the O–H stretches from water, and the first overtone of the N–H stretch from the amide groups in collagen. This region is highly sensitive to both hydration levels and the integrity of the bone’s protein structure.
- around 1720 nm (Scanner): first overtone of C–H stretching vibrations. As with the 1200 nm band, this signal may come from both collagen and lipids.
- around 1940 nm (Scanner): combination of O–H stretching and H–O–H bending vibrations. The intensity of this band, along with the one at around 1450 nm, may provide an indication of the sample’s water content.
- around 2050 nm and around 2180 nm (Scanner): they are attributed to combination bands of the amide groups (involving N–H and C=O bonds) that form the protein’s peptide backbone. They may serve as direct indicators of the presence and state of the collagen matrix.
- 2300–2350 nm (Scanner): this complex region corresponds to combination bands involving C–H stretching and bending vibrations. It may be associated with lipids but also may receive a significant contribution from collagen.

**Figure 2:**
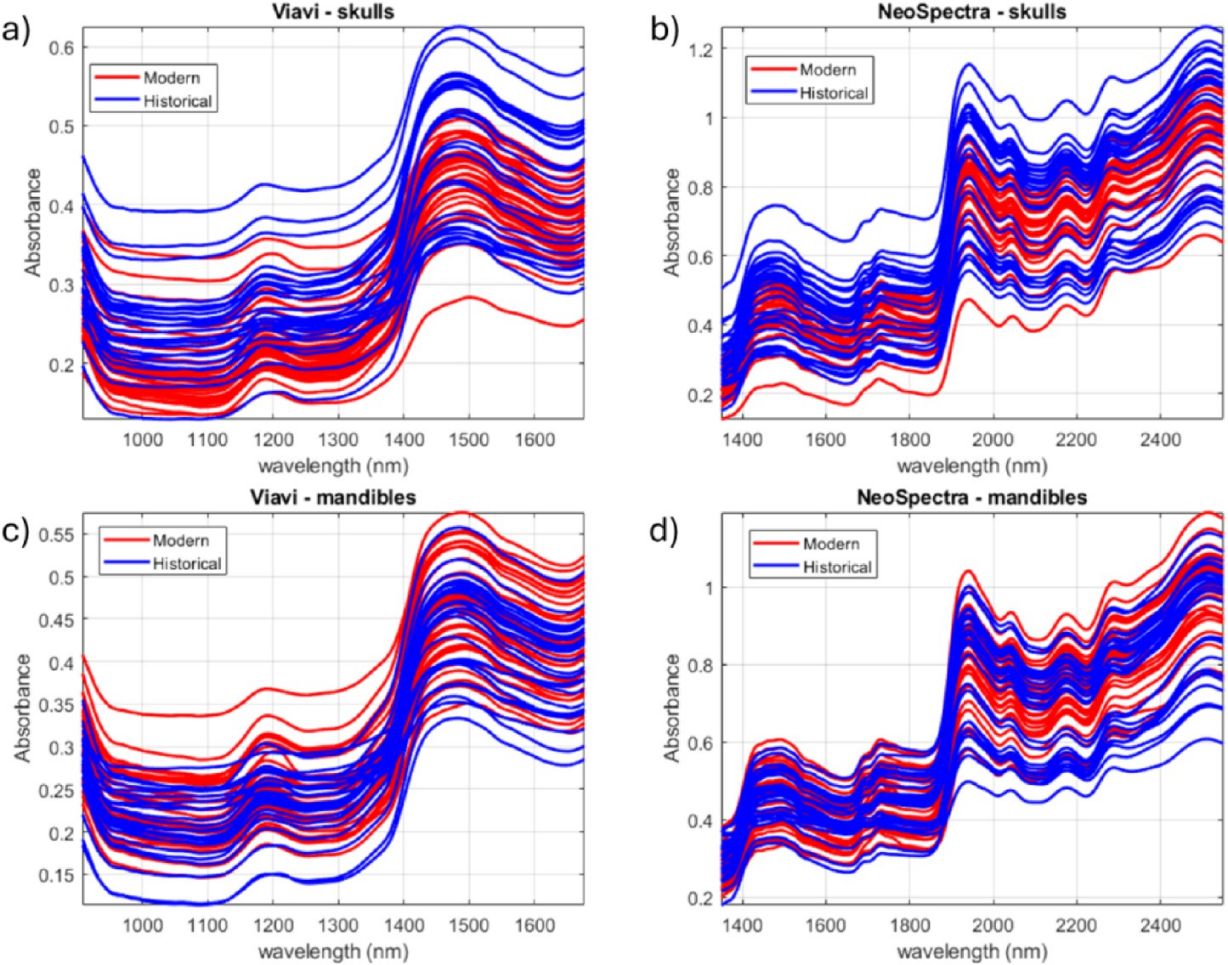
Spectra of the samples analyzed. a) skulls analyzed with MicroNIR, b) skulls analyzed with Scanner, c) mandibles analyzed with MicroNIR, d) mandibles analyzed with Scanner.

Comparing plots [Fig fig2]a and 2b (skulls) with plots [Fig fig2]c and 2d (mandibles), the overall spectral features are very similar, which is expected as both are bone tissues with the same underlying composition. 

Historical skull samples generally exhibit higher absorbance values across nearly the entire spectral range compared to modern skulls, particularly in the main water absorption bands around 1450 and 1940 nm. Although one might expect historical bones to be drier, this increased absorbance may result from diagenetic changes. Specifically, postmortem degradation can enhance bone porosity, enabling the bone to absorb and retain more environmental moisture. This pattern, however, is less evident for mandible samples.^53^ A similar pattern of elevated absorbance in historical skulls is also observed in regions associated with the lipidic fraction. This is consistent with known diagenetic transformations affecting lipids in bone marrow.^54^

### Study of Multivariate Error

Figure 3 shows the error covariance and error correlation matrices for the skull and mandible data from both instruments. For enhanced clarity, each matrix is displayed with both a main plot and a corresponding top-down view.

**Figure 3:**
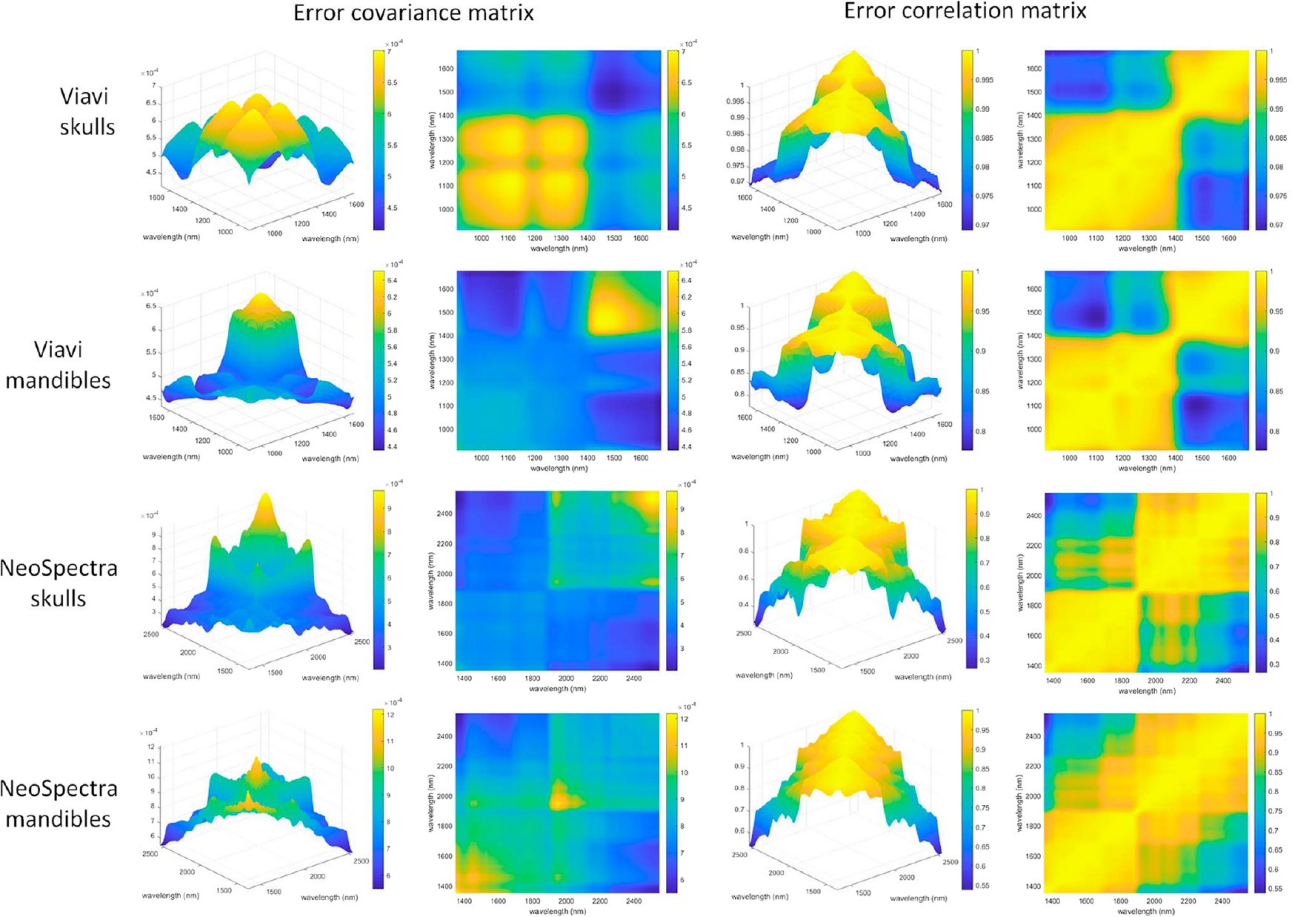
Error covariance matrices and error correlation matrices (in columns, together with a top-down view of the corresponding matrices) for skulls, mandibles and the two sensors used.

Observing the error covariance matrices in Figure 3 (the first column shows a 3D representation of each ECM, while the second column provides a top-down 2D view of the same matrices), it can be seen that the shape of the ECMs for the skull and mandible analyzed with the same instrument are different. For this reason, when calculating the classification models, we opted to build separate models for the skull and the mandible for each instrument, rather than combining the data to create a single ECM and a model that included both sample types. Regarding the magnitude of the errors, the ECMs shows that the errors are similar for the Scanner and the MicroNIR (when analyzing both skulls and mandibles), but higher in the case of the Scanner.

Figure 3 provides significant insights into the nature of the measurement error. The nonuniform shape of the diagonal in the ECM plots demonstrates that the error is not constant, but rather wavelength-dependent. The most critical observation comes from the error correlation matrices, which display large, off-diagonal blocks of high correlation. This structured pattern suggests the presence of multiplicative error and indicates that the noise is not random white noise. Instead, it suggests the presence of systematic effects, such as variations in light scattering due to sample texture or positioning, or instrumental drift, which cause entire spectral regions to fluctuate in a correlated manner. The fact that the correlation matrix is entirely positive is also indicative of the presence of scattering.^32^

Measurement error often increases with signal intensity, so the differences in mean absorbance spectra between skulls and mandibles lead to distinct error patterns. Additionally, the physical properties of the bones—such as surface roughness, density, and porosity—differ between the flatter cranial bones and the more complex mandibles, affecting light scattering and contributing to variability. The results indicate that the sample type influences both the magnitude and structure of the errors.

### Classification Strategy and Best Classification Models Obtained

As is common in chemometric analysis, an exploratory PCA was initially performed. In the resulting PCA score plots no separation between the two classes emerged; even after applying various preprocessing methods, some overlap between the classes was observed. Therefore, we decided to incorporate the multivariate error information into the subsequent data analysis through implementation of MLPCA. The use of MLPCA was also motivated by the need for a tool to properly handle multivariate instrumental data, such as spectra. In such data, the foundational PCA assumption of independent and identically distributed (iid) errors is often not valid.

MLPCA can be understood as a generalization of PCA that explicitly incorporates the ECM into its framework. The fundamental principle of MLPCA is to effectively disentangle the true variance/covariance structure of the underlying variables from the measurement noise by optimally leveraging prior knowledge of the error structure. It is important to note that the validity of MLPCA still relies on the assumptions that the underlying model is linear and that the dimensionality of the subspace is correctly identified.

A significant practical advantage of this approach is that the application of MLPCA can often eliminate the need for complex and sometimes poorly understood data preprocessing methods.^55,56^ Much of the motivation for preprocessing multivariate data has been to transform the measurement errors to be more uniform and uncorrelated, thereby making the data more compliant with the iid assumptions required for PCA. MLPCA provides a more rational alternative by directly modeling the known error structure rather than attempting to correct it.

A further significant distinction between PCA and MLPCA lies in the estimation of subspace models with varying dimensionalities. For PCA, the solutions are nested, meaning a model with fewer components can be obtained by simply truncating a higher-dimensional model. Conversely, MLPCA solutions are not nested; each subspace model must be calculated independently. For instance, the two-component MLPCA model could be not equivalent to the first two components of a three-component model. This necessity to recompute the entire model for each dimensionality increase the computational cost, particularly when numerous models of different ranks must be evaluated. In this study, as described below, models until 5 components were computed and analyzed. Furthermore, there are other crucial distinctions between the two methods. PCA projects data onto the subspace using an orthogonal projection, whereas MLPCA employs a maximum likelihood projection,^57^ which exploits the measurement error information to obtain the best possible estimate of the true, noise-free values.

Although MLPCA was first described in 1997,^35^ its application in the scientific literature has remained relatively limited, arguably not reflecting its theoretical advantages, likely due to practical and conceptual barriers. Key among these is somewhat higher conceptual and computational complexity compared to standard PCA, its limited availability in mainstream statistical software packages, and the practical need to estimate a reliable error covariance matrix from experimental data, which collectively raise the threshold for its implementation.

A critical decision in the methodology is how to incorporate measurement error variance and covariance into the MLPCA data decomposition, as this choice determines which MLPCA algorithm (from cases A to F) is subsequently employed. In this study, a globally pooled ECM, derived from all specimens—both historical and modern—was applied to every sample. This required the use of the MLPCA algorithm D for model computation.^37^ Alternative strategies were considered but discarded. Using individual ECMs per sample, based on only five replicates, would be unreliable and require many more measurements, making it costly and time-consuming. Using class-specific ECMs for historical and modern specimens would complicate predictions, as assigning the correct ECM to a new sample would require knowing its class in advance, which is precisely what the model aims to determine.

Figure 4 shows the score plots for the two-PC MLPCA models, calculated from Scanner and MicroNIR data for the analysis of skulls and mandibles using the whole data set.

**Figure 4:**
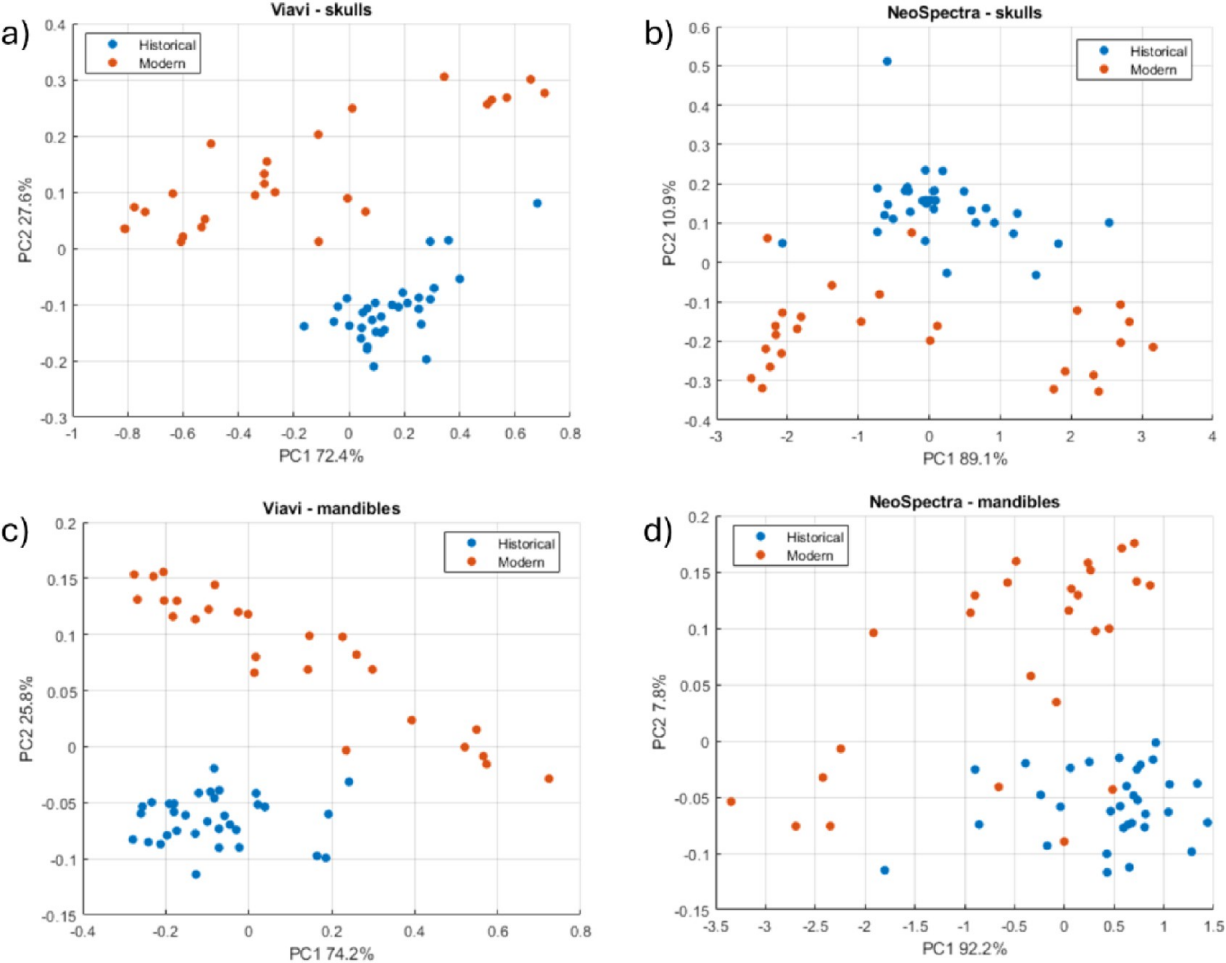
Score plots corresponding to the two-PC MLPCA models for a) MicroNIR and skulls; b) Scanner and skulls; c) MicroNIR and mandibles; d) Scanner and mandibles.

The MLPCA models achieve a good separation between the two classes of samples since MLPCA is specifically designed to account for noniid error structures, though its effectiveness relies on an accurate estimation of the ECM.

Following the dimensionality reduction with MLPCA, the resulting sample scores were subsequently used as predictor variables to build a classification model based on LR. This two-step MLPCA-LR approach combines a robust method for dimensionality reduction with a powerful and interpretable classification framework.

The selection of LR for the classification stage was justified by several key advantages for this binary problem (classification of two classes: historical and modern bones). Foremost, LR is a direct probabilistic classifier, fundamentally designed to model the probability of class membership, providing not only a definitive class assignment but also a valuable measure of certainty for each prediction. As a discriminant technique, LR is also explicitly optimized to find a clear decision boundary that best separates the two classes. Crucially, the use of LR is particularly appropriate in this context because the scores from the MLPCA model serve as ideal input variables: a small set of information-rich, uncorrelated predictors that circumvent the primary limitations of applying regression models to highly collinear spectroscopic data. Furthermore, the computational cost of the proposed strategy is minimal, with a runtime of approximately 1–2 s per model.

MLPCA-LR models with up to five PCs were constructed for the analysis of skulls and mandibles using the Scanner and MicroNIR data sets on the training set. The regression models were validated using cross-validation. For each data set and sample type, the optimal model was selected by balancing high classification performance with the minimum number of PCs, while carefully avoiding overfitting. Performance parameters were calculated by defining class 1 (“historical” bones) as the positive class, and the resulting optimal models are summarized in Table 1.

**Table 1:** Quality parameters for the best MLPCA-LR models obtained using cross-validation on the training set^a^

	PCs	Accuracy	Sensitivity	Specificity	Precision	NPV	FPR	FNR	AUC
MicroNIR skulls	2	1.00	1.00	1.00	1.00	1.00	0.00	0.00	1.00
Scanner skulls	2	1.00	1.00	1.00	1.00	1.00	0.00	0.00	1.00
MicroNIR mandibles	2	0.95	0.91	1.00	1.00	0.90	0.00	0.09	0.99
Scanner mandibles	2	0.87	0.90	0.83	0.86	0.88	0.17	0.10	0.83

a The column ‘PCs’ refers to the number of PCs selected in the corresponding MLPCA model. The table reports standard classification parameters: accuracy, sensitivity (true positive rate), specificity (true negative rate), precision (positive predictive value), negative predictive value (NPV), false positive rate (FPR), false negative rate (FNR), and the area under the curve (AUC).

The classification results demonstrate a very high performance for the two components MLPCA-LR models, especially in the analysis of skulls. For the analysis of skulls, both the MicroNIR and Scanner instruments yielded perfect classification models, with all performance parameters reaching their optimal values of 1. A differentiation in performance emerges, however, when analyzing the mandibles. The model built with MicroNIR data maintained a very high classification capability, achieving an accuracy of 0.95 and an excellent AUC of 0.99. Notably, it retained perfect specificity and precision (1.00), indicating no false positives, though a minor drop in sensitivity (0.91) was observed. In contrast, the Scanner model for mandibles exhibited a more considerable decrease in performance, with an accuracy of 0.87 and an AUC of 0.83. This reduction was primarily driven by lower specificity (0.83) and precision (0.86), resulting in a false positive rate of 0.17. This disparity suggests that achieving a clear separation between the two classes is more challenging when analyzing the mandibles compared to the skulls, with the MicroNIR spectrometer proving more robust for this difficult classification task. Although both instruments perfectly classified skulls, Scanner’s performance (specificity, AUC) dropped markedly on mandibles, unlike MicroNIR’s. This discrepancy may be attributed to differences in optical design, possibly due to its spectral range (1350–2550 nm), potentially omitting key absorption features in the lower NIR region (970–1200 nm) which MicroNIR captures.

The observed decrease in classification performance for mandibles may attributable to their greater anatomical and compositional heterogeneity compared to skulls. Mandibles possess a complex morphology, combining dense cortical bone with porous trabecular bone, particularly in the alveolar region.^58^ This structural variability, along with their unique geometry, may introduce higher variance into the spectral data due to inconsistent light scattering and challenges in achieving reproducible probe contact during measurement, an issue exacerbated by their smaller size, which often results in incomplete coverage of the instrument’s measurement window. Furthermore, this inherent porosity could facilitate a more heterogeneous diagenetic process over time, leading to greater intraclass spectral variation among historical samples.^59^ Collectively, these factors would render the classification of mandibles an inherently more complex task. It should also be noted that the measuring window of both sensors is relatively large compared to the size of the mandibular samples, which may contribute to the lower performance observed.

Figure 5 shows the loadings for the four MLPCA models shown in Table 1. Interpreting the MLPCA loadings is essential for uncovering and understanding the information captured and rationalized by the model.^60^ The first principal component (PC1) in all four plots is hypothesized to primarily model physical features arising from factors like inconsistent sample coverage and the resulting light scattering effects. These physical features manifests in the loadings as a broad, baseline-like feature. This baseline could be modulated by the primary NIR absorption bands of water present in their respective ranges (around 1450 and 1940 nm), suggesting a partial leakage of the most dominant chemical signal into the first component.

**Figure 5:**
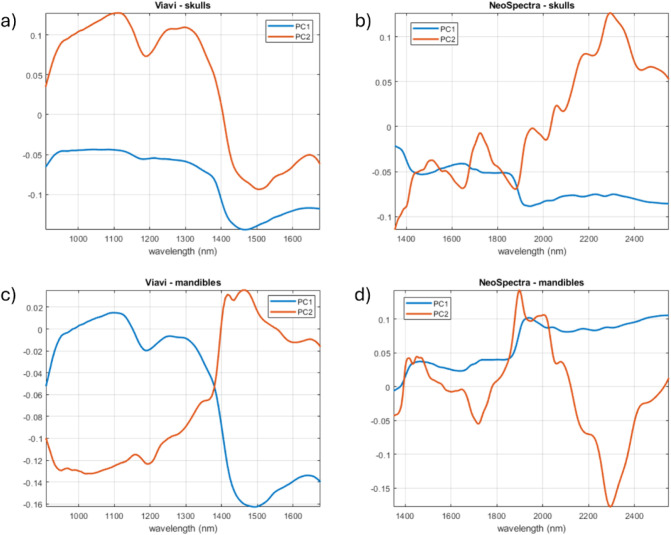
Loading plots of the four MLPCA models selected using the MLPCA-LR classification strategy: a) MicroNIR and skulls; b) Scanner and skulls; c) MicroNIR and mandibles; d) Scanner and mandibles.

Following the isolation of these effects, the second principal component (PC2) in all four models appears to capture the most significant chemical information related to bone composition. The loading plots for PC2 exhibit features consistent with the known NIR absorbances of bone’s main constituents. Specifically, the loadings show prominent features that can be tentatively assigned to water (around 1450 and 1940 nm) and the organic protein matrix, collagen (e.g., around 1200 nm, 1500 nm, 1720 nm, and in the 2050–2350 nm region).

Table 2 presents the external validation (test set) results for the optimal MLPCA-LR models. Three of the four models demonstrated outstanding generalization. The models for skulls (both MicroNIR and Scanner) and the mandible model based on the MicroNIR spectrometer achieved flawless classification, with all performance metrics reaching optimal values. In contrast, the model for mandibles using the Scanner instrument exhibited a drop in performance. While it maintained a high sensitivity (0.91), its overall accuracy was only 0.79. The primary weakness was a specificity of 0.63, leading to a high FPR of 0.38. This indicates that the model tends to misclassify negative samples as positive. The low AUC of 0.66 further confirms its weak discriminative power on this challenging data set.

**Table 2:** Performance parameters of the optimal MLPCA-LR models, obtained by applying models trained on the training set to the external test set^a^

	PCs	Accuracy	Sensitivity	Specificity	Precision	NPV	FPR	FNR	AUC
MicroNIR skulls	2	1.00	1.00	1.00	1.00	1.00	0.00	0.00	1.00
Scanner skulls	2	1.00	1.00	1.00	1.00	1.00	0.00	0.00	1.00
MicroNIR mandibles	2	1.00	1.00	1.00	1.00	1.00	0.00	0.00	1.00
Scanner mandibles	2	0.79	0.91	0.63	0.77	0.83	0.38	0.10	0.66

a The column ‘PCs’ refers to the number of PCs selected in the corresponding MLPCA model.

Comparing the test set predictions with the initial CV results provides insight into the models’ robustness and generalizability. For the skulls, the perfect scores obtained in CV for both sensors were perfectly mirrored in the test set results, confirming the development of highly robust and generalizable models. A similar coherence was observed for the model of mandibles using the MicroNIR instrument. The excellent performance seen during CV was fully corroborated by the perfect classification achieved on the test set. This consistency between CV and external validation underscores the reliability of this model. Conversely, the Scanner mandibles model showed a discrepancy between CV and test performance, indicating a lack of generalization. The performance metrics degraded across the board from CV to the test set: accuracy dropped from 0.87 to 0.79 and specificity fell from 0.83 to 0.63, and the AUC decreased from 0.83 to 0.66. This degradation suggests that the model developed for this combination of sensor and sample type is not robust enough to perform reliably on new, unseen samples.

The robustness of the classification models was also rigorously assessed using a bootstrap validation method with 1000 iterations on the training set, with the results presented in Table 3. The bootstrap procedure was performed on the training set. This comprehensive validation provides a realistic and stable estimate of the overall performance and robustness of the four classification models.

**Table 3:** Performance metrics for the optimal MLPCA-LR models from bootstrap validation, calculated using 1000 iterations on the entire data set^a^

	PCs	Accuracy	Sensitivity	Specificity	Precision	NPV	FPR	FNR	AUC
MicroNIR skulls	2	0.95 (0.08)	0.99 (0.06)	0.93 (0.12)	0.94 (0.11)	0.98 (0.09)	0.07 (0.12)	0.01 (0.06)	0.998 (0.013)
Scanner skulls	2	0.92 (0.09)	0.95 (0.10)	0.91 (0.13)	0.92 (0.12)	0.93 (0.13)	0.09 (0.13)	0.05 (0.10)	0.99 (0.03)
MicroNIR mandibles	2	0.93 (0.09)	0.97 (0.10)	0.93 (0.12)	0.93 (0.11)	0.95 (0.12)	0.07 (0.12)	0.03 (0.09)	0.998 (0.012)
Scanner mandibles	2	0.79 (0.16)	0.88 (0.20)	0.74 (0.19)	0.79 (0.16)	0.85 (0.21)	0.26 (0.19)	0.13 (0.20)	0.84 (0.14)

a The reported values represent the mean of the 1000 iterations, with the corresponding standard deviation shown in parentheses. The ‘PCs’ column indicates the number of principal components used in the MLPCA model. Significant figures are shown according to ref 61.

The analysis reveals that the models developed for skulls with both instruments and for mandibles with the MicroNIR instrument are highly effective and reliable. These three models consistently show high mean accuracies (ranging from 0.92 to 0.95) and excellent discriminative power, with AUC values of 0.99 or higher. In contrast, the model for Scanner mandibles demonstrates weaker performance, with a mean accuracy of 0.79 and an AUC of 0.84. Its primary limitation appears to be a low mean specificity (0.74), resulting in a high false positive rate (0.26). Furthermore, the standard deviations from the bootstrap resampling provide critical insight into model stability. The three high-performing models (analysis of skulls with MicroNIR, analysis of skulls with Scanner and analysis of mandibles with MicroNIR sensors) all exhibit high robustness, as evidenced by their low standard deviations across all metrics (e.g., AUC standard deviation ≤0.03). This indicates that their performance is stable and not overly dependent on the specific samples chosen for training. Conversely, the Scanner mandibles model is shown to be unstable. It displays larger standard deviations for all parameters (e.g., accuracy std. dev. = 0.16; AUC std. dev. = 0.14). This variability signifies that the model’s performance is sensitive to the training data composition, making it somewhat less consistent for practical application.

When comparing these bootstrap results to those from CV and the external test set (Tables 1 and 2), a slight difference in performance is apparent. Both the CV and the external test set validation demonstrate exceptionally high, often perfect, performance for the skulls (both instruments) and the analysis of mandibles using the MicroNIR instrument, with accuracies frequently reaching 1.00. The bootstrap validation, which offers a more robustly averaged assessment, reflects a similar pattern of high performance for these three models (e.g., mean accuracies between 0.92 and 0.95, and mean AUCs > 0.99). All validation methods consistently identified the model built on Scanner sensor mandible data as the weakest, showing lower and more variable performance across all metrics. Therefore, the bootstrap validation provides a more conservative estimate of performance compared to cross-validation or a single test set. This discrepancy arises from their distinct validation schemes. By using sampling with replacement and out-of-bag (OOB) evaluation, the bootstrap ensures that every sample—including the most challenging ones—serves as a test sample multiple times across the iterations. Consequently, the final averaged score smooths out the stochasticity of any single data partition, providing a more realistic and robust estimate of the model’s true generalization performance.

### Insights from Results: A Comparison with Classical PLS-DA

For comparative purposes, the performance of the MLPCA-LR models was compared to that of a more classical classification approach, PLS-DA. The first and essential step in building the PLS-DA classification models was the application of the optimal preprocessing to the data, ensuring its error structure was as close as possible to the iid assumption.^62^ Conventionally, the optimal preprocessing is selected by evaluating how a model’s performance metrics vary as a function of the number of latent variables and the specific preprocessing applied.^55,63^ To select the optimal preprocessing for the PLS-DA classification models, we used the visual error-matrix strategy we had introduced in an earlier publication.^64^ This approach provides insight into the key data characteristics and aims to identify the preprocessing pipeline that renders the data most compliant with the iid error assumption required by PLS-DA. This compliance is visually assessed through the error correlation matrix, which for ideal iid data would exhibit constant values on the diagonal and zero-values in the off-diagonal elements.

Figure 6 illustrates the changes in the error correlation matrix after applying the optimal preprocessing combination selected for each data set. The selected preprocessing strategies were as follows (all data were subsequently mean-centered for PLS-DA modeling):

**Figure 6:**
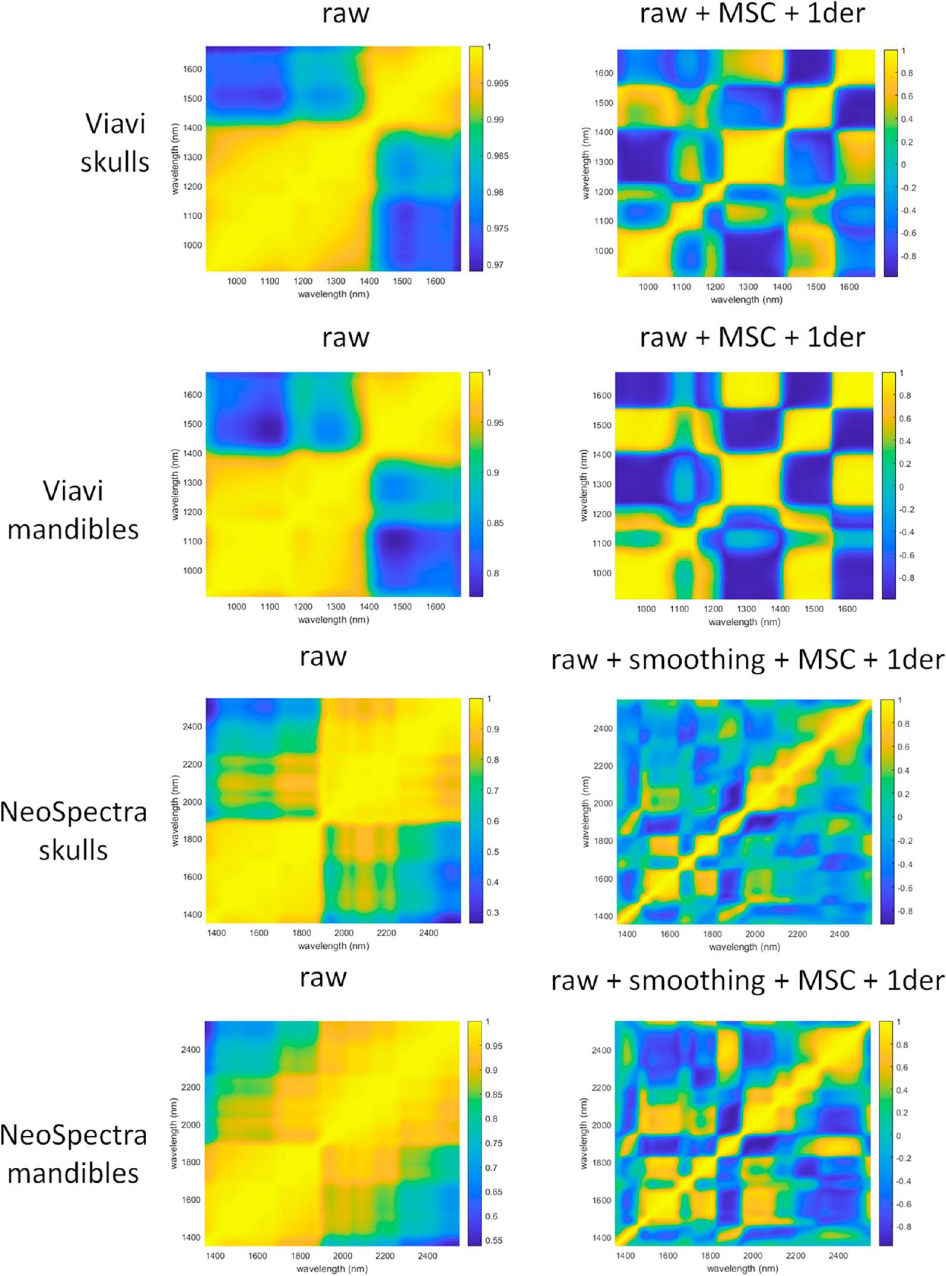
Error correlation matrix for skulls, mandibles and the two instruments used, with the best combination of preprocessing methods applied.

MicroNIR skulls: MSC + first derivative

Scanner skulls: Smoothing (15 points) + MSC + first derivative

MicroNIR mandibles: MSC + first derivative

Scanner mandibles: Smoothing (19 points) + MSC + first derivative

As observed in the Figure 6, the preprocessing reduces the correlation between errors in the off-diagonal values. While the raw data errors exhibited a highly structured pattern, the preprocessed data errors appear more random. This randomization of the error structure, however, is achieved to varying degrees depending on the instrument and sample type. Although perfect iid conditions were not fully achieved, this transformation makes the data structure more suitable for PLS-DA modeling, which ideally assumes a random error distribution.

Table 4 shows the performance metrics for the optimal PLS-DA classification model for each case, presenting the model performance from both cross-validation on the training set and prediction on the external test set. While the PLS-DA models provided effective classifications, they generally did not achieve the same level of performance as the MLPCA-LR models. The performance of the PLS-DA models was more variable between cross-validation and the test set. Furthermore, the MLPCA-LR models often achieved their superior performance using an equal or smaller number of components, indicating greater efficiency and robustness.

**Table 4:** Quality parameters for the best PLS-DA models^a^

	LVs		Accuracy	Sensitivity	Specificity	Precision	NPV	FPR	FNR	AUC
MicroNIR skulls	3	CV	0.98	1.00	0.94	0.96	1.000	0.06	0.00	0.99
		Test	0.83	0.90	0.75	0.82	0.86	0.25	0.10	0.98
Scanner skulls	4	CV	0.93	0.91	0.94	0.95	0.89	0.06	0.09	0.99
		Test	1.00	1.00	1.00	1.00	1.00	0.00	0.00	1.00
MicroNIR mandibles	4	CV	0.95	0.95	0.94	0.95	0.94	0.06	0.05	0.98
		Test	0.95	0.91	1.00	1.00	0.89	0.00	0.09	1.00
Scanner mandibles	4	CV	0.87	0.95	0.78	0.83	0.93	0.22	0.05	0.95
		Test	0.79	0.82	0.75	0.82	0.75	0.25	0.18	0.77

a The column ‘LVs’ refers to the number of latent variables selected in the corresponding PLS-DA model.

A fundamental distinction between the two methods lies in their approach to data variance. The performance of PLS-DA is critically dependent on the selection of an optimal data preprocessing strategy, a process that can be complex and nontrivial. The MLPCA-LR framework, conversely, obviates the need for such preprocessing. It is specifically designed to model the error directly within the model structure, although its success is contingent upon an accurate estimation of the ECM. This inherent ability to separate the relevant information from the error structure appears to provide a significant advantage for this particular classification problem.

## Conclusions

This study demonstrates that portable NIR spectroscopy combined with an MLPCA-LR framework offers a rapid, nondestructive, and accurate approach for distinguishing historical and modern bones. The models achieved near-perfect classification for both skulls and mandibles, although mandibles proved more challenging due to their greater anatomical heterogeneity and peculiar shape, which should be considered in future analyses. The proposed approach is not intended to replace precise dating performed by established reference techniques or domain experts, but rather to assist in the rapid classification of large numbers of samples into chronological categories, with models calibrated on specimens of known age enabling the classification of essentially unlimited numbers of similar samples.

A key advantage of MLPCA-LR over conventional PLS-DA lies in its ability to directly incorporate the measurement error structure, reducing the need for complex and subjective data preprocessing, while the interpretation of loadings provided insights into chemical variations in water and collagen content as drivers of classification, reflecting postmortem degradation. This methodology is therefore practical and robust, particularly for museum collections, as its combination of portable, low-cost NIR instruments and a straightforward protocol allows rapid screening of specimens by nonexpert personnel.

Beyond museum applications, NIR spectrometry holds strong potential for forensic and conservation contexts, including classification of CITES (Convention on International Trade in Endangered Species of Wild Fauna and Flora) specimens or recovery of historically significant biological materials, unlocking the research potential of large zoological collections that remain largely unexplored. Overall, the results suggest that, although the model developed is currently applicable within the specific geographical context investigated, the proposed methodology appears generally valid and capable of discriminating samples according to their chronological attribution, with broader applicability primarily depending on the availability of additional data sets.

## Supplementary Material



## Notes

Photographs of various archeological samples analyzed in this study (PDF)
