# Supplementary material for: Shedding Light on the Past: Temporal Classification of Zoological Specimens from Museum Collections with Portable NIR Sensors and Multivariate Error Modeling
Source: Anal Chem. 2026 Mar 20;98(13):9658–71. doi: 10.1021/acs.analchem.5c06767 (PMC13063218; doi:10.1021/acs.analchem.5c06767)
Supplement: Supplementary file 1 [file ac5c06767_si_001.pdf]

Classification of zoological specimens into historical periods using portable NIR sensors and multivariate error modeling

Jordi Riu[1], Barbara Giussani*[2], Manuel Monti[2], Lorenzo Baruffaldi[2], Marc Campeny[3], Javier Quesada[3]

[1] Universitat Rovira i Virgili. Department of Analytical Chemistry and Organic Chemistry. Carrer Marcel·lí Domingo 1, 43007 Tarragona, Spain

[2] Dipartimento di Scienza e Alta Tecnologia, Università degli Studi dell'Insubria, Via Valleggio 9, 22100 Como, Italy

[3] Museu de Ciències Naturals de Barcelona, Castell dels Tres Dragons, Passeig Picasso s/n, 08003 Barcelona, Spain

Figures S1–S5 display various archaeological samples analyzed in this study.

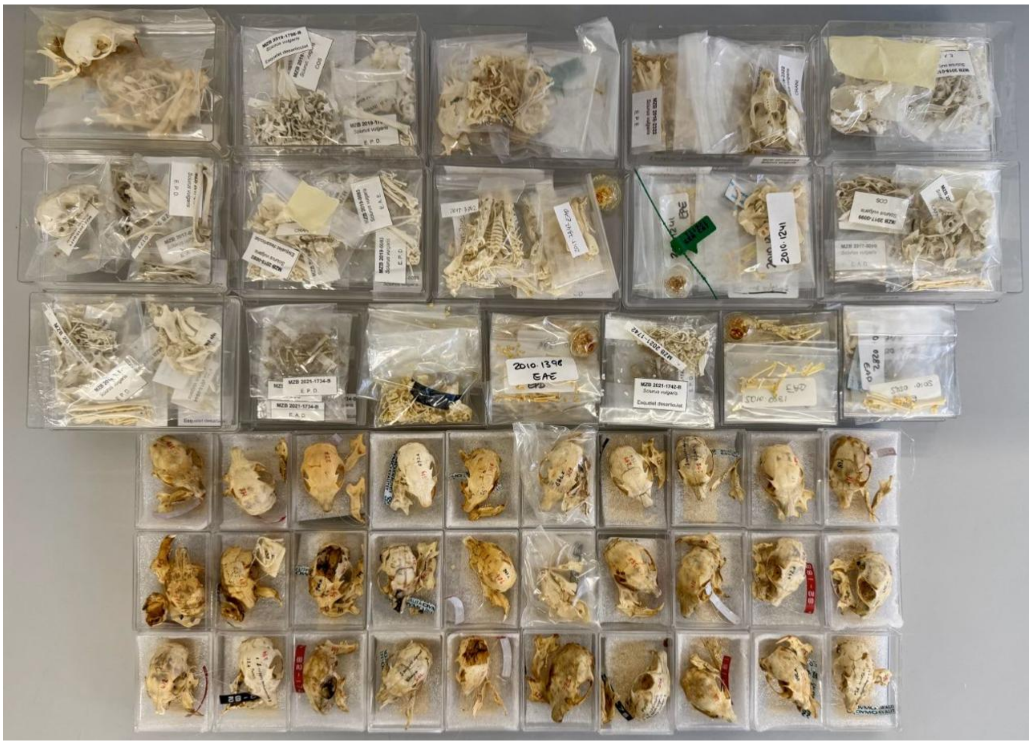

Figure S1. Overview of the samples analyzed in the study.

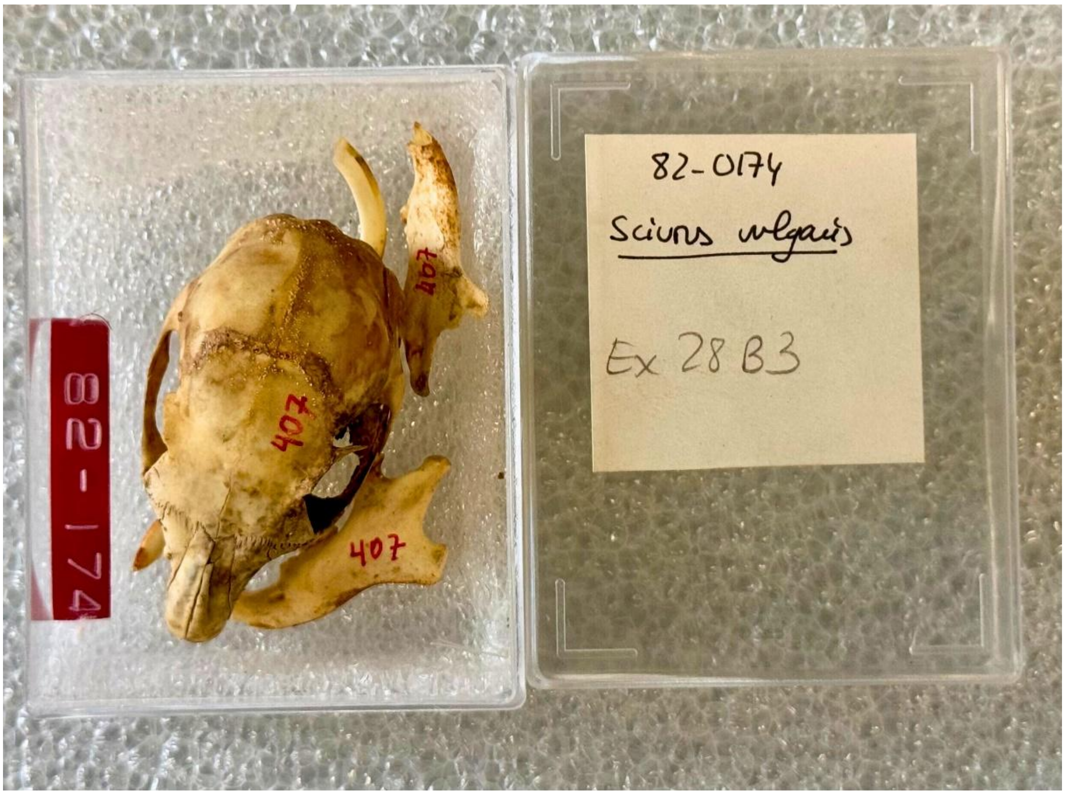

Figure S2. Example of a historical sample analyzed in this study.

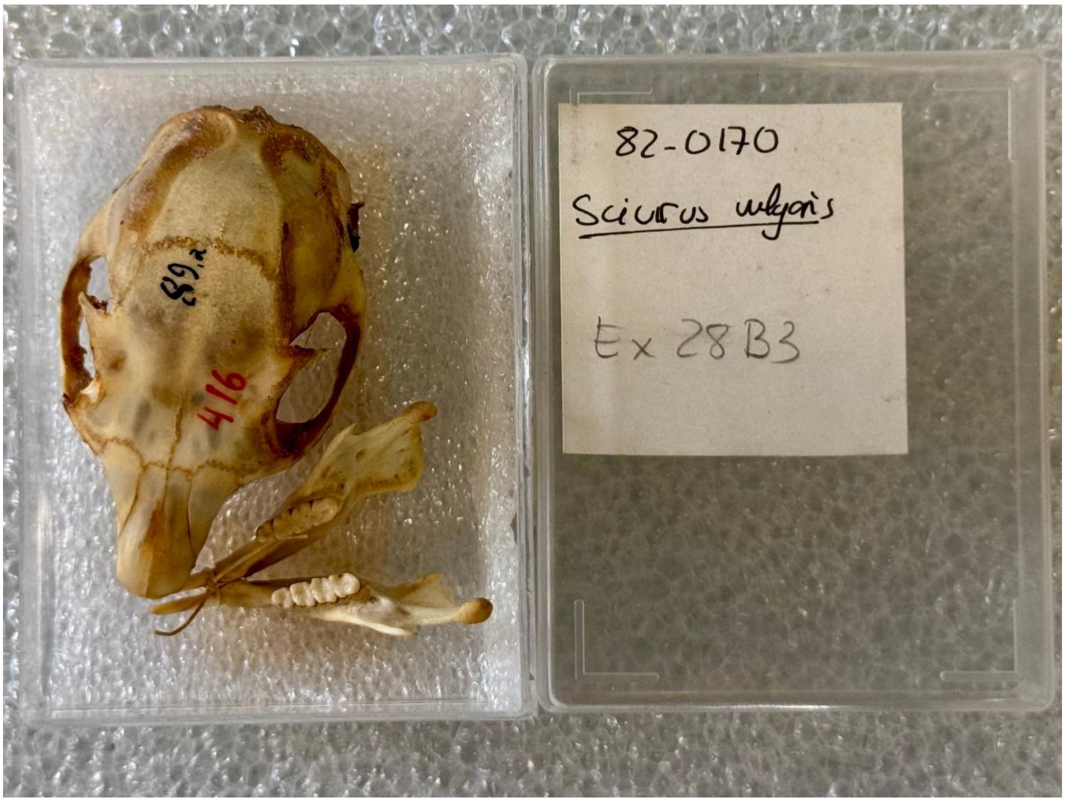

Figure S3. Example of a historical sample analyzed in this study.

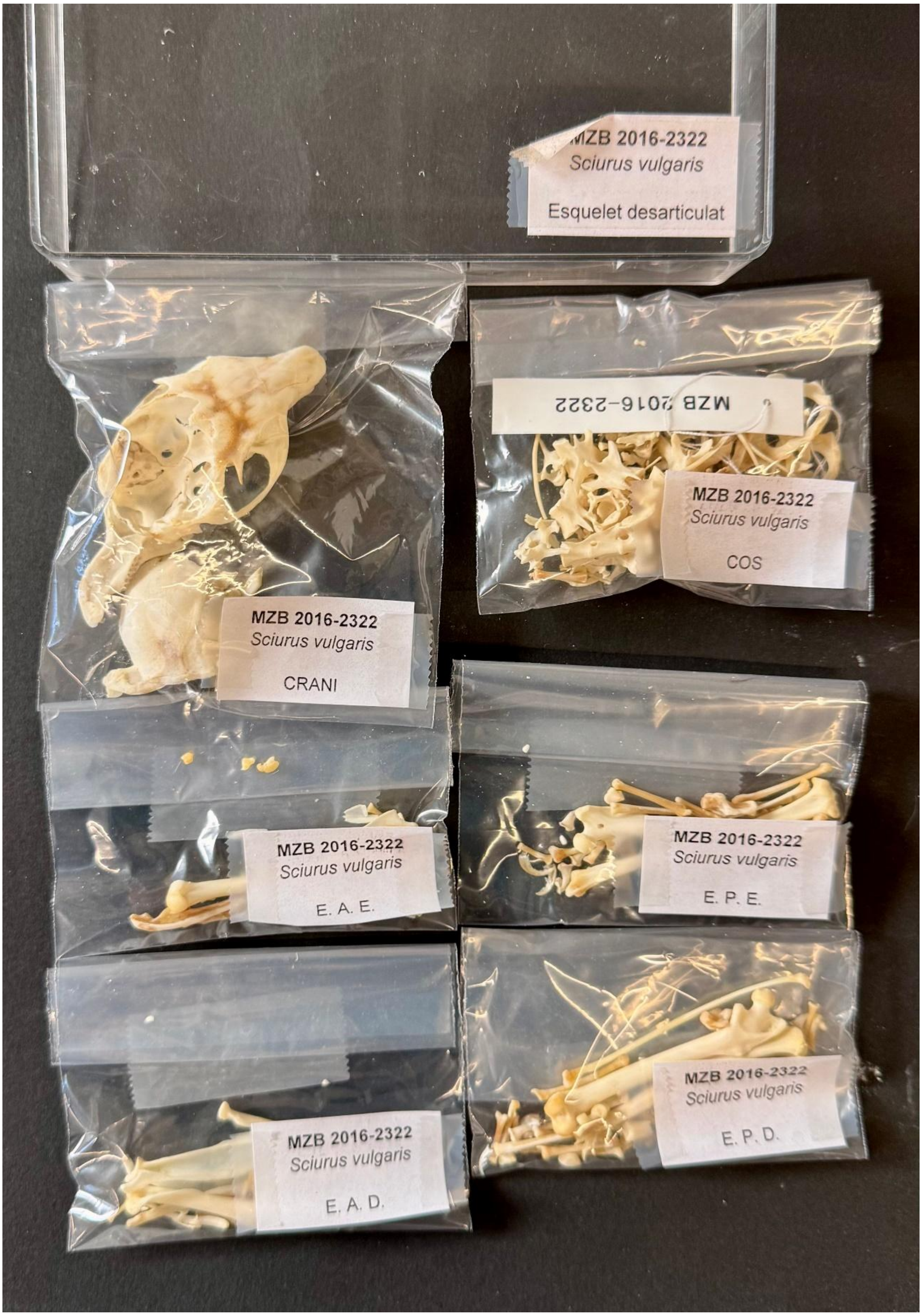

Figure S4. Example of modern samples analyzed in this study.

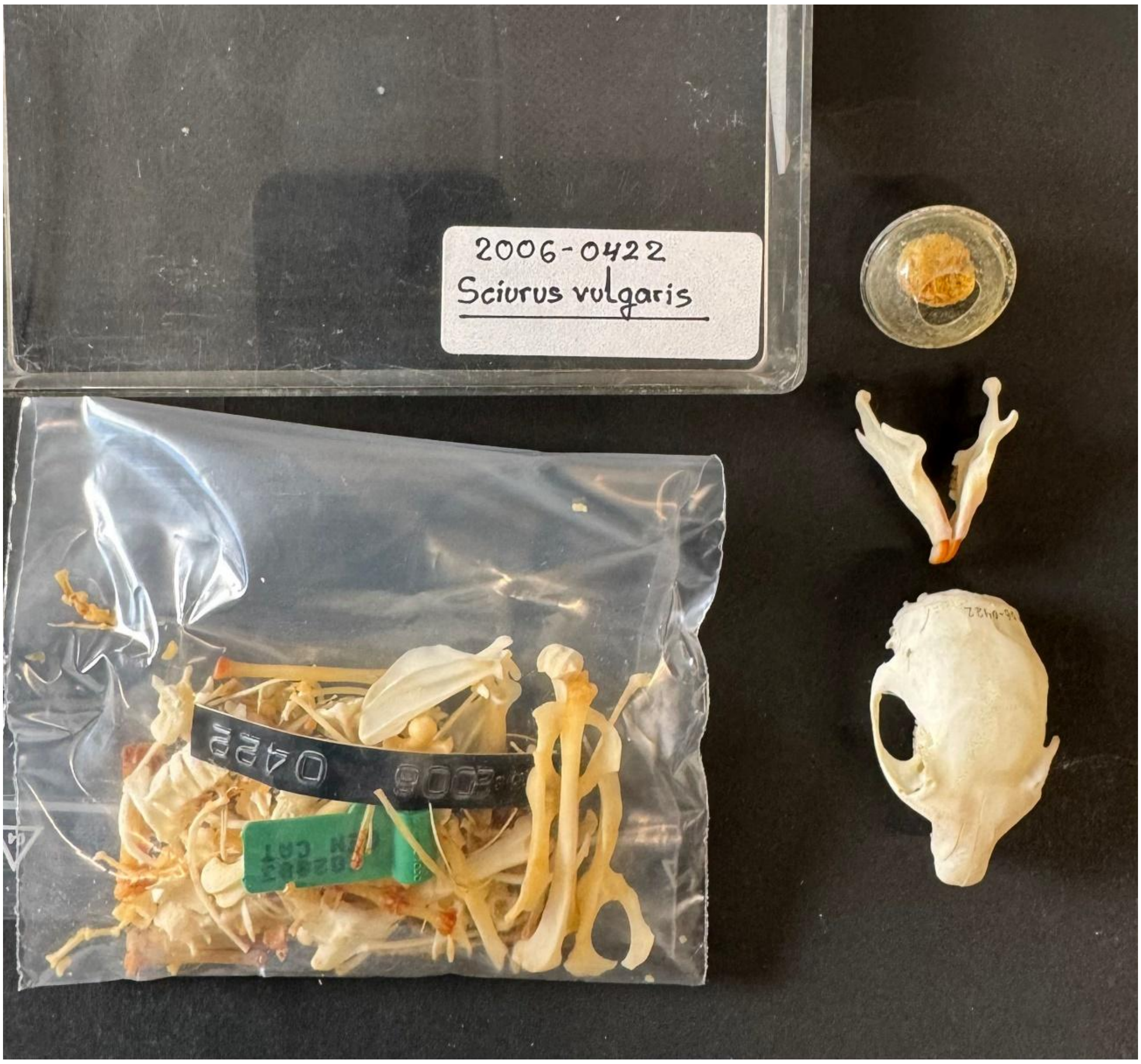

Figure S5. Example of modern samples analyzed in this study.

Figures S6 and S7 show the measurement process using the NeoSpectra and Viavi.

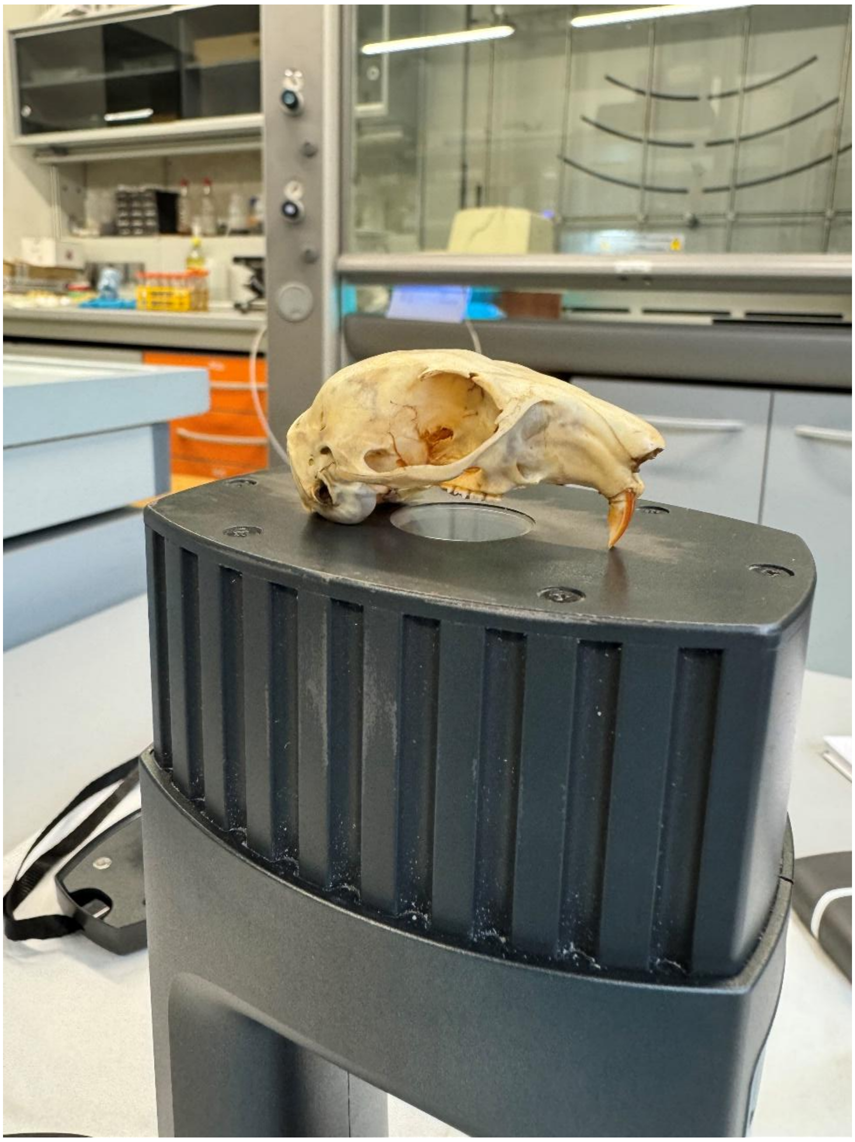

Figure S6. Measurement process using the NeoSpectra.

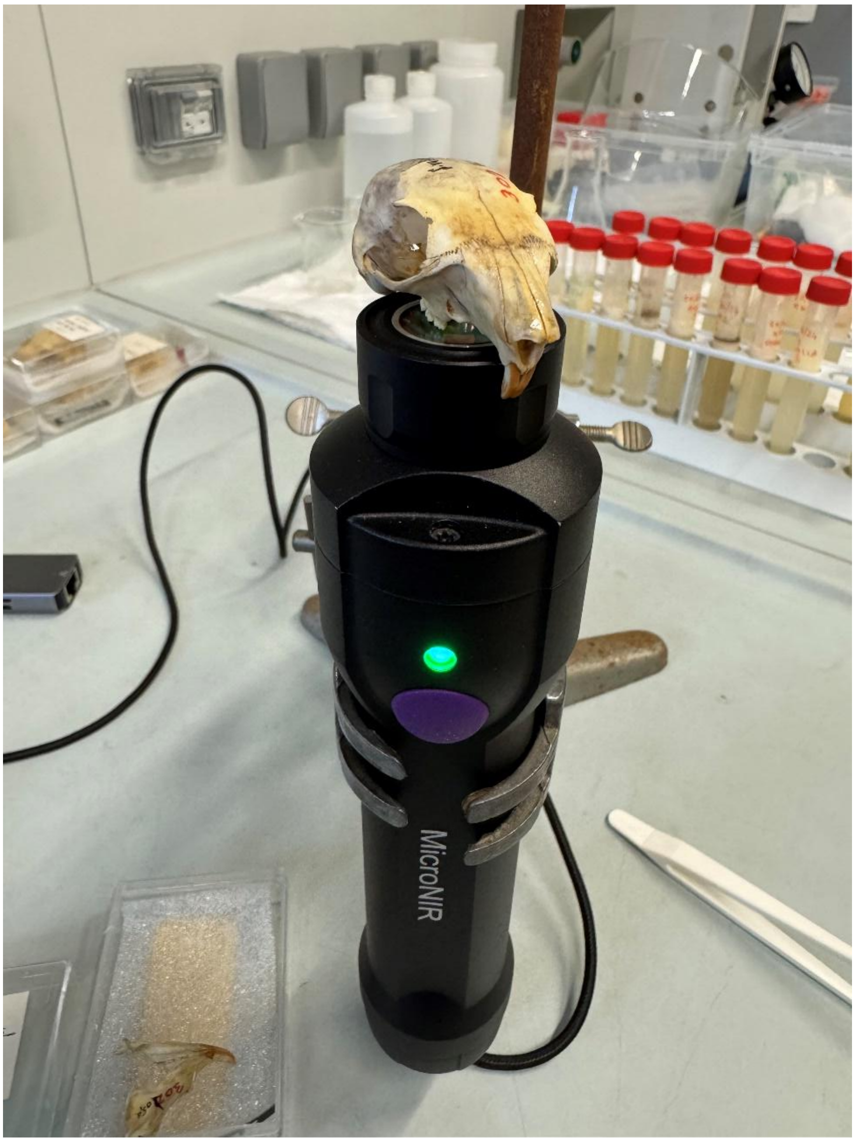

Figure S7. Measurement process using the Viavi.
